# Analysis of Regional Variation in the Scope of Eligibility Defined by Ages in Children's Medical Expense Subsidy Program in Japan

**DOI:** 10.3389/fphar.2017.00525

**Published:** 2017-08-22

**Authors:** Takuma Sugahara

**Affiliations:** Faculty of Economics, Hosei University Tokyo, Japan

**Keywords:** medical expense subsidy, regional variation, socio-economic factors, instrumental variables, ordered logit, co-payments, income limits

## Abstract

Children's medical expense subsidy programs are programs run by local governments that use public monies to reduce or eliminate the copayments for children's medical treatment including pharmaceutical cost (typically 20% for preschoolers and 30% thereafter). Currently, all prefectures and municipalities in Japan provide subsidies for infants' and children's medical expenses, but scope on ages of eligibility, income limits, and copayment requirements vary. The fact that these programs are run by local governments has given rise to differences in the costs borne by households with children, depending on the jurisdiction in which they live. Therefore, although it would be desirable to gain society's understanding of such variation, the factors have not been fully studied. This analysis investigates what factors could impact such variation. In it, we looked at 219 municipalities in the prefectures in the Kanto region, focusing on the gap from the average age eligibility of municipalities, which reflects the scope of eligibility. Neither a regression analysis using the instrumental variable method to account for simultaneous decision bias nor an ordered logit analysis with rank of coverage as an order variable revealed that differences in copayments by locale had any impact on the scope of age eligibility. Residents' income and the number of children tended to narrow scope of eligibility for subsidies, but the strength of local government finances were not a significant factor of influence. In designing these programs, local government bodies take into account the local population's ability to pay and the number of eligible people, but their awareness of the local government's financial condition seems to be scant. Local governments are currently moving to expand their children's medical expense subsidy programs, but in the future they will need to pay more attention to balancing an expanded scope of eligibility by ages with the maintenance of local government fiscal discipline. In addition, copayments have not been adequately linked to the expansion of eligibility, so it would be advisable to clearly demonstrate the reason for this limit in order to eliminate perceptions of unfairness.

## Introduction

### Background and objectives

Under Japan's public health insurance system, copayments that the insured persons pay to the medical institutions and pharmacies for doctor visits and drugs are basically 30 and 20% for children before the age of compulsory schooling. Children's medical subsidy programs are those run by independent local governments which reduce or eliminate the copayments paid when children visiting medical institutions (20% for pre-compulsory education children, 30% thereafter) and are funded by the public funds of each municipality. With regard to this subsidy system in Japan, each prefecture sets their own scope by ages for coverage in children's medical expense subsidy at first. And second, based on the prefectural standard, each municipality within the prefecture sets the scope of the coverage by ages in addition to the prefectural scheme to extend the coverage for the residents. And within this scope of coverage, Children can basically enjoy their medical service at free of charge, even if it was rather tiny copayment. As a result, there exists variation of scope of coverage by ages depending on the municipalities.

Currently such programs are in place in all prefectures and municipalities, according to the FY2015 “Survey of Aid for Medical Expenses for Infants and Children” issued by the Equal Employment, Children and Families Bureau of the Ministry of Health, Labour and Welfare (MHLW). The survey shows that in the largest number of municipalities, aid is available “up to junior high school students” for both “inpatients” and “outpatients”; however, differences exist among the municipalities with respect to “age coverage,” “existence or non-existence of income limits,” and the “existence or non-existence of copayment.”

Advantages cited in the past for the implementation of medical assistance programs include “elimination of unfairness in medical benefits due to economic disparities,” “economic support for the child-rearing generation,” and “reduction of aggravated cases due to refraining from visiting the doctors.” In line with this context, in the United States, some researcher clearly shows that positive correlation between health status of children and socio-economic status (Case et al., 2002; Currie and Stabile, 2003; Condliffe and Link, 2008). Buchmueller et al. (2005) suggest that health coverage surely has effect to increase the number of outpatient visits, and Currie et al. (2008) shows to have this type of health subsidy system contribute to improve the health status.

On the other hand, disadvantages cited for this system include “encouraging competition among the municipalities for the child-rearing generation,” “concerns about the possible increase of unnecessary hospital visits” (Bessho, 2011), and “concerns that families will think less about their children's health.”

Out of a concern of the potential that the independent assistances by municipalities may encourage easygoing medical consultations, the national government has historically followed “measures to reduce the national health insurance” with respect to medical expenses increases in stemming from the assistance programs (Iwamoto, 2010).

Since the children's medical subsidy programs are basically locally-run programs, they also have given rise to variation in the costs borne by the child-rearing families depending on the local jurisdictions they live in. The factors driving these variation have not yet been fully examined, notwithstanding the desirability of gaining some degree of public understanding.

With that in mind, in this study, we focused on the variation of scope of coverage defined by ages and tried to address the factors that have (or do not have) significant effect to the coverage. This study examined the impact of various program designs and socio-economic factors of 219 municipalities in five prefectures of Kanto Area (Kanagawa, Saitama, Chiba, Tochigi, and Ibaraki), focusing in particular on the “the gap from the average age eligibility of the municipalities for subsidy” that reflects the scope of the children's medical subsidy programs. We look at medical subsidy for “outpatient” visits, where there is a greater variation among locales than for “inpatient treatment.”

## Materials and methods

### Data sources

We obtained information about, the “scope of coverage for subsidy defined by ages,” “existence or non-existence of tiny copayments at the time of medical use,” and the “existence or non-existence of income limits at the time of registration,” that define the nature of eligibility and the scope for subsidy from the “Operational Status of Publicly Funded Programs for Medical Expenses for Infants and Children” issued by the Ministry of Health, Labour and Welfare: Maternal and Child Health Division of the Equal Employment/Children and Families Bureau (2015). This report is annually published and opened on the websites by Children and Families Bureau of the MHLW. This report gathers information about the current status of Children's Medical Expense Subsidy Program, that completely covers all municipalities in Japan and enough trustworthy. We also consulted the websites of each local municipality in addition to confirm the nature of their subsidy programs to be sure (in weeks 3 and 4 of July, 2016). But there was very few information difference between these two data resources besides the gap of investigation timing.

As for the socio-economic factors on each municipalities that might impact to the scope of coverage defined by ages, we gathered information from “Population by Age Groups” (Ministry of Internal Affairs and Communications, 2015a); “Population Ratios by Age Groups” (Ministry of Internal Affairs and Communications, 2015a); “Per-capita Medical Expenses” (Ministry of Health, Labour and Welfare, 2015); “Average Incomes” (prepared by author from Ministry of Internal Affairs and Communications, 2015b); and “Fiscal Capability Index” (Ministry of Internal Affairs and Communications, 2015b).

### Background of hypothesis

In this study, we assumed that the scope of coverage defined by ages for children's medical subsidy is decided by each local government based on the prefectural-level operations of the program. And that is the reason why the scope of coverage defined by ages has variation among municipalities. It is also natural to assume that “the gap from the average age eligibility of the municipalities for subsidy” has variation among municipalities and this is largely affected by not only programmatic factors but socio-economic factors.

Based on these ideas, we established the following working hypotheses. As for setting tiny copayments required for subsidy, leaving tiny copayments in place to a certain degree, rather than eliminating them altogether, holds back excessive doctor visits and reduces fiscal burden, freeing up additional fiscal resources which could enable expansion of age eligibility requirements. On the other hand, if a tiny copayment is the proxy expressing a strict stance on the part of local government leaders toward medical subsidy, requiring copayments at the time of medical use has a negative effect on age eligibility. So the expected *a priori* direction of impact on “the gap from the average age eligibility of the municipalities for subsidy” is not so clear in advance.

As for setting income limits for registration of subsidy, restricting the subsidy only to those with incomes under certain limit narrows eligibility with the effect of lightening the cost to the local government, thereby freeing up additional fiscal resources and potentially enabling expansion of scope of age eligibility requirements. On the other hand, if setting the income limit is the proxy expressing a strict stance on the part of local government leaders toward medical subsidy, income limits have a negative effect on age eligibility. Therefore, the expected *a priori* direction of impact is not also clear.

With regard to socio-economic factors, we considered following four factors [i.e., Average income of local residents, Local government fiscal strength, Per capita resident medical expense, and Child (ages 0–14) population and its ratio] that might have some effect to the gap from the average age eligibility of the municipalities for subsidy.

As for the case with average income of local residents, local governments with residents of higher average incomes, meaning the residents have greater ability to pay for medical services themselves, will establish narrower scope for medical subsidy programs for children than the other municipalities. To the contrary, local governments in strong fiscal health can take on additional costs, allowing them to provide expanded government services to their residents, and will thus establish broader scope for medical subsidy programs for children than the other areas. And local governments with higher per capita medical expenses will be concerned about fiscal impact and as such establish narrower scope for medical subsidy programs for children than the others. Of course the greater the absolute number of local children eligible for the program, and the greater the ratio of children in the total population, the greater the concern about the cost to the local government, which will lead to narrower scopes for children's medical subsidy programs than the other municipalities.

### Method of data analysis

Based on hypotheses, we took as mainly explained variable the “the gap from the average age eligibility of the municipalities for subsidy” and performed a regression analysis with programmatic factors and socio-economic factors as explanatory variables. We first did an ordinary least-square (OLS) estimation with all programmatic factors and socio-economic factors as explanatory variables.

Looking at the distribution of “age limits for coverage” in medical subsidy programs for children, we note that the frequencies are concentrated at ages 12, 15, and 18. We therefore created an order variable with a value of 1 for the upper age of 12 as the narrow scope of coverage, a value of 2 for upper age of 15 as middle scope of coverage, and a value of 3 for upper age of 16 and older as wide scope of coverage and also conducted the ordered logit model estimation with this order variable.

When determining the applicable scope of ages to which subsidy is to be extended in comparison with the status of the other areas, other programmatic factors such as the “establishment of partial copayments” and “income limits” might be considered simultaneously. To deal with this problem, we use the instrumental variable method. In our case, it is required that the instrumental variables be related to the explanatory variables of “copayments” and “income limits,” but not to factors impacting the “the gap from the average age eligibility of the municipalities for subsidy.” After careful investigation of instrumental variable including endogenous test (Durbin-Wu Hausman Test) and weak correlation test (2Stage Least Square size of nominal 5% test), I found and adopted “Ibaraki(Prefecture) Dummy” as satisfying instrumental variable. It has significant correlation to both “Copayment” and “Income Limit” but does not have any relation to “the gap from the average age eligibility of all the municipalities” that is finally decided by each municipalities. Considering the heteroskedastic problem among cluster on prefecture, we adopted cluster robust regression. We also check the correlation among the variables in advance and investigate VIF (the Variance Inflation Factor) to avoid multicollinearity problem in our estimation.

## Results

The basic statistics for the dataset and variable names in the estimation are shown in Table 1. Although the dataset we built for this analysis was limited to the five prefectures in Kanto Area, in comparisons with nationwide data, the ratios of “existence or non-existence of partial copayment for subsidy” and “existence or non-existence of income limit for subsidy” were both nearly identical with those of the national data [Children and Families Bureau of the Ministry of Health, Labour and Welfare (2015)].This table shows that the rate of municipalities that is setting any copayment occupy just 40% and Income limit setting is 21% in Kanto Areas respectively. Population ratio of under14 years is now 12% in Kanto areas nearby Tokyo. The correlation among the variables used in the estimation model is summarized in Table 2. We can see the existence of strong correlation between financial status of local government and average household income of residents.

**Table 1:** Basic statistics of data set.

**Variable**	**Obs**	**Mean**	**Std. dev**.	**Min**	**Max**
**DEPENDENT VARIABLES**					
Gap from the Average Age eligibility: **GAPAVE**^a^	219	0.00	1.80	−6.05	4.94
Order Category on Rank of Coverage: **ODRCATG** (1,2,3)	219	2.04	0.52	1.00	3.00
**INDEPENDENT VARIABLES**					
Copayment setting for Subsidy: **COPAY** (No = 0/Yes = 1)	219	0.40	0.49	0.00	1.00
Income Limit setting for Subsidy: **INCMLIM** (No = 0/Yes = 1)	219	0.21	0.41	0.00	1.00
The Number of Population Under14Years :**POP014**	219	16273.67	39282.99	244	481532
Population Ratio Under14 Years: **RATIO014**	219	0.12	0.02	0.07	0.17
Financial Status of Local Government: **FINANIDX**	219	0.76	0.22	0.20	1.50
Medical Expenditure per Capita/year: **MEDEXP**^b^	219	293391.70	20618.55	237495.40	356550.90
Average Household Income: **AVEINCM**^c^	219	298.42	35.15	233.22	434.48
Saitama Prefecture Dummy: **SAITDUM** (No = 0/Yes = 1)	219	0.29	0.45	0.00	1.00
Chiba Prefecture Dummy: **CHIBADUM** (No = 0/Yes = 1)	219	0.25	0.43	0.00	1.00
Kanagawa Prefecture Dummy: **KANADUM** (No = 0/Yes = 1)	219	0.15	0.36	0.00	1.00
Tochighi Prefecture Dummy: **TOCHDUM** (No = 0/Yes = 1)	219	0.11	0.32	0.00	1.00
Ibaraki Prefecture Dummy: **IBARDUM** (No = 0/Yes = 1)	219	0.20	0.40	0.00	1.00

a *Average Age Eligibility of Municipalities = 15.05*.

b *1 Japanese Yen*.

c 10,000 Japanese Yen;

*1 dollar = 117.5 Yen in 2016 (in Average)*.

**Table 2:** Correlation among variables.

**(obs = 219)**	**GAPAV**	**COPAY**	**INCMLIM**	**POP014**	**RATIO014**	**FINANIDX**	**AVEINCM**	**MEDEXP**
Gap from the Average Age eligibility: **GAPAV**	1							
Copayment setting for Subsidy: **COPAY**	0.1909	1						
Income Limit setting for Subsidy: **INCMLIM**	−0.5239	0.1666	1					
The Number of Population Under14Years : **POP014**	−0.3264	−0.065	0.1877	1				
Population Ratio Under14 Years: **RATIO014**	−0.1652	−0.0405	0.0459	0.2119	1			
Financial Status of Local Government: **FINANIDX**	−0.2407	−0.1695	0.0411	0.2813	0.6633	1		
Average Income: **AVEINCM**	−0.3858	−0.14	0.1907	0.4297	0.6337	0.7214	1	
Medical Expenditure per Capita/year: **MEDEXP**	−0.1921	−0.3486	0.0116	−0.0366	−0.4001	−0.1669	−0.0835	1

Table 3 shows the estimates from the regression model using “the gap from the average age eligibility of all the municipalities” and “order category on rank of coverage” as the explained variable and the programmatic factors and socio-economic factors of each local government as explanatory variables. In Table 3, model (1) and (2) are the results of simple OLS estimations performed without regard to simultaneity of system determination. Models (3) and (4) give the estimates from the ordered logit model we ran with the explained ordered variable. In these estimation, average household income (AVEINCM) and medical expenditure per capita (MEDEXP) was continuously statistically significant as negative effect to expanding scope of coverage. In simple OLS model, the number of population under 14 years old (POP014) was also significant, suggesting that the number of child in the municipalities might have effect to narrow the scope of coverage of subsidy for children.

**Table 3:** Regression results on simple OLS model, Ordered logit and instrumental variable method.

**(Number of Obs = 219)**	**Simple OLS regression**		**(Number of Obs = 219)**	**Ordered logit regression**		**(Number of Obs = 219)**	**Instrumental variable method**	
	**Dependent variable**	**Gap from the Average Age eligibility GAPAV**		**Dependent variable**	**Order Category on Rank of Coverage ODRCATG**		**Dependent variable**	**Gap from the Average Age eligibility GAPAV**
	**(1)**	**(2)**		**(3)**	**(4)**		**(5)**	**(6)**
Copayment setting for subsidy	0.245		Copayment setting for Subsidy	0.389		Copayment setting for Subsidy	0.080	
**COPAY**	(1.06)		**COPAY**	(1.17)		**COPAY**	(0.30)	
Income Limit setting for subsidy		−1.9816^***^	Income limit setting for subsidy		−4.0310^***^	Income limit setting for subsidy		0.145
**INCMLIM**		(−6.43)	**INCMLIM**		(−5.44)	**INCMLIM**		(0.28)
The number of population under 14 Years	−0.00001^***^	−0.00000661^***^	The number of population under 14 years	−0.00000587	−0.00000309	The number of population under 14 years	−0.00000897^***^	−0.00000917^***^
**POP014**	(−3.85)	(−4.01)	**POP014**	(−1.26)	(−0.82)	**POP014**	(−8.58)	(−5.54)
Population ratio undue 14 Years	−2.6702	−6.0157	Population ratio undue 14 Years	−5.311	−10.4960	Population ratio under 14 Years		
**RATIO014**	(−0.34)	(−0.88)	**RATIO014**	(−0.40)	(−0.72)	**RATIO014**		
Financial status of Local Government	0.3467	−0.4004	Financial status of local government	0.3497	−0.5761	Financial status of local government	0.181	0.192
**FINANIDX**	(0.54)	(−0.70)	**FINANIDX**	(0.36)	(−0.54)	**FINANIDX**	(0.18)	(0.19)
Average household income:	−0.016^***^	−0.0093^***^	Average household income:	−0.0270^***^	−0.0219^***^	Average household income:	−0.017^***^	−0.017^***^
**AVEINCM**	(−3.90)	(−2.65)	**AVEINCM**	(−3.96)	(−2.84)	**AVEINCM**	(−3.52)	(−2.67)
Medical expenditure per Capita/year	−0.00002^***^	−0.0000209^***^	Medical expenditure per Capita/year	−0.0000248^**^	−0.0000326^***^	Medical expenditure per capita/year	−0.000019^***^	−0.000020^***^
**MEDEXP**	(−2.58)	(−3.77)	**MEDEXP**	(−2.50)	(−3.03)	**MEDEXP**	(−3.07)	(-2.44)
_cons	10.295^***^	10.483^***^				_cons	10.548^***^	10.908^***^
	(4.52)	(5.54)					(4.71)	(3.15)
F(6, 212)	11.34	22.85	Wald chi2(6)	47.94	59.78	Wald chi2(5)	316.98	387.37
Prob > F	0.00	0.00	Prob > chi2	0.00	0.00	Prob > chi2	0.00	0.00
R-squared	0.24	0.42	Pseudo R2	0.15	0.34	Pseudo R2	0.23	0.20
Root MSE	1.59	1.38	Log likelihood	−141.89	−111.32	Log likelihood	1.568	1.600
Mean VIF	1.93	1.88						

In model (1) and (2): t value in parentheses: ^***^t < 0.01, ^**^t < 0.05. HCSE, Heteroscedasticisty-consistent standard errors are used.

In model (3) and (4): z value in parentheses: ^***^z < 0.01, ^**^z < 0.05.

In model (5) and (6): z value in parentheses: ^***^z < 0.01, ^**^z < 0.05. Instrumental variable: Ibaraki Prefecture Dummy Cluster Robust Standard errors are used.

Model (5) and (6) give the estimates employing the “Ibaraki prefecture Dummy” as instrumental variable. As previously described, we tried endogenous test and weak correlation test (Stock and Yogo, 2005) to check appropriateness of instrumental variable. In these estimations, not only AVEINCM and MEDEXP but also POP014 were significant with negative sign.

## Discussion and conclusion

Japan's overall population is already on the downward trend, with the number of aged increasing rapidly and the number of births dropping. Pushing forward with measures to deal with the declining birthrate is not only one of the pillars of government policy, but for local governments also an urgent challenge on which their continued existence depends. In this context, the topic of medical subsidy programs for children, as one element of child-rearing support, is a matter of high interest, but being managed by local governments, such programs differ in their eligibility details.

Our results indicate that for medical subsidy programs for children leaving in place some kind of copayments within the program was not a clear effect factor on the scope of age eligibility of subsidy. While such program designs might well have the effect of reducing doctor visits, not to mention easing the financial burden on the local government, they appear to have little impact on expanding the scope of eligibility of the subsidy programs. If, as our estimates suggest, the establishment of copayments do not lead to any expansion of eligibility which would benefit residents even when keeping constant the income level of residents and local government fiscal status, local governments establishing such limits need to explain more clearly to residents the reasons and basis for establishing them. In general, income limits has the effect to narrow scope of age eligibility, which means that the introduction of income limits may be proxy of strict financial stance of policy maker.

With regard to local socio-economic factors, we showed that “AVEINCM” is likely to have the effect of narrowing the scope for age eligibility. This suggests that local governments consider the household income status of local residents in designing the programs. From the perspective of economic bearing ability, local governments with more residents with relatively high incomes have relatively less need to provide unconditional subsidy for medical consultations for children, and in that context, this can be considered a reasonable result. Having said that, income disparities in Japan have become more prominent in recent years, meaning that it is not necessarily appropriate to gauge the economic circumstances of residents by average income alone. Whether or not the magnitude of income disparities within a local government entity affects the scope of eligibility for assistance is an important issue, especially given the overall intent of medical assistance programs for children, which is to provide necessary medical care to children in the area regardless of income level, but due to our data limitations we were unable to incorporate that into the current analysis; we hope to be able to address that issue sometime in the future.

We also showed that both “medical expenses per capita (MEDEXP)” and “The number of population under14 years (POP014)” in the locale had a negative effect on scope for subsidy, in line with our hypothesis. It is understandable that jurisdictions with high per capital medical expenses would be greatly concerned about ballooning medical expenses and show a tendency to narrow the eligibility scope. The number of children in a jurisdiction is a factor determining the number of recipients of subsidy. It is also understandable from the perspective of financial burdens on the government entity, that to the extent the eligible population is greater, the government would tend to want to narrow the eligibility scope for subsidy.

Contrary to our *a priori* expectations, the financial status of local government (FINANIDX) did not have a statistically significant effect on the scope of eligibility of medical subsidy programs for children. One research preceding ours and made at the prefectural level did not demonstrate a clear relationship between the fiscal soundness of the municipality and the income limits, but it has been found that copayments tend to be reduced in prefectures with higher indices of fiscal soundness (Nishikawa, 2010). Conceivable reasons that indices of fiscal soundness had no effect on the scope of eligibility include (1) an increasing perception on the part of local governments that medical subsidy programs for children should be put in place regardless of budgetary considerations, given the fact that child-rearing support is currently a top-priority being pushed by the central government; (2) local residents demand implicitly that their government provide support at least equivalent to that of neighboring entities when the latter expand the scope of their subsidy programs; and (3) medical subsidy programs for children are a mainstay of regional child-rearing support policies, serving as featured policies to attract the child-bearing generation, and should be put in place independent of present financial circumstances. Previous study suggests that there exists some relationship between the timing of the election and the reform of subsidy system (Nishikawa, 2011). Although child-rearing support is unquestionably a critical policy for Japan, discussions must be deepened going forward about to what extent, given the strong demands on local governments for fiscal discipline, should public funds be allocated to medical subsidy programs for children, and to what extent should such public funding be provided by central vs. local governments.

In the current children's medical expense subsidy programs, municipalities consider the expected expenditure size and ability to pay of residents when setting up the system. On the other hand, sufficient consideration has not been given to the financial situation of the municipalities themselves, and there must be a serious problem from the viewpoint of fiscal discipline. In addition, setting of some copayment and income limits have not influenced the scope of coverage even when other factors are fixed (ceteris paribus), and from the view of fairness of the burden of medical expenses among residents of different regions, it is necessary to reconsider its relevance.

This analysis addresses just five prefectures in Kanto Area. In the future it will be necessary to confirm the reliability of our results by expanding our analysis to other areas of Japan.

## Author contributions

The author confirms being the sole contributor of this work and approved it for publication.

### Conflict of interest statement

The author declares that the research was conducted in the absence of any commercial or financial relationships that could be construed as a potential conflict of interest.
